# Draft Genome Sequences of *Buttiauxella* spp. Isolates from Water and Gastropods with Putative β-d-Glucuronidase Activity

**DOI:** 10.1128/mra.00064-22

**Published:** 2022-03-02

**Authors:** Carolin Leister, Michael Hügler

**Affiliations:** a TZW: DVGW-Technologiezentrum Wasser, Karlsruhe, Germany; University of Maryland School of Medicine

## Abstract

We report the draft genome sequences of *Buttiauxella* spp. strains that were isolated from water and gastropods. Three isolates show fluorescence in the Colilert system, indicating unusual β-d-glucuronidase activity, and phylogenetic analyses suggest that they represent a novel species. Another strain, without β-d-glucuronidase activity, was assigned to the species Buttiauxella ferragutiae.

## ANNOUNCEMENT

Microbial water quality is examined using fecal indicator bacteria such as coliform bacteria and Escherichia coli. For their detection, β-d-galactosidase and β-d-glucuronidase activities are tested with membrane-filtration-based water quality tests or most probable number methods like the Colilert system (1, 2). E. coli is the most important indicator of fecal water contamination, and the presence of β-d-glucuronidase activity is considered indicative of E. coli (2, 3). However, other members of the *Enterobacteriaceae* family, such as certain strains of Salmonella, Klebsiella, *Citrobacter, Shigella*, and *Yersinia*, also possess this enzyme, resulting in false-positive E. coli results (3–5). Here, we report draft genome sequences of *Buttiauxella* isolates that showed false-positive E. coli results in water analyses.

*Buttiauxella* spp. strains were isolated from a drinking water sample from a small village near Bruchsal, Germany, and from feces from the gastropods Arion vulgaris and Helix pomatia, collected near Bruchsal (Table 1), using the Colilert-18/Quanti-Tray (IDEXX Laboratories, USA) according to ISO 9308-2:2012 (6). To obtain single colonies, liquid from the wells of the Colilert-18/Quanti-Tray was transferred onto heterotrophic plate count (HPC) agar plates (Merck KGaA, Darmstadt, Germany) (7), as recommended by German regulations (Deutsches Einheitsverfahren), and incubated for 24 h at 36°C. Bacterial isolates were picked, transferred to fresh HPC agar plates, and again incubated for 24 h at 36°C. Genomic DNA of pure cultures grown on these agar plates was extracted using the FastDNA SPIN kit for soil (MP Biomedicals, USA) and quantified using a Qubit fluorometer (Invitrogen, USA) according to the manufacturer’s instructions.

**TABLE 1:** Characteristics and accession numbers of genomes from *Buttiauxella* spp.

Bacterial species	Strain	Sample	Sampling date (yr-mo-day)	*uidA*	No. of reads	Genome coverage (×)	Genome size (bp)	No. of contigs	*N*_50_ (bp)	G+C content (%)	No. of coding sequences	GenBank accession no.	SRA accession no.
*Buttiauxella* sp.	S04-F03	*Arion vulgaris*	2017-06-06	+	592,495	36.3	4,799,994	36	310,867	50.1	4,365	JAJSOS000000000	SRR17259675
*Buttiauxella* sp.	W03-F01	*Helix pomatia*	2017-06-06	+	556,906	34.1	4,785,902	25	398,126	50.0	4,350	JAJSOT000000000	SRR17259674
*Buttiauxella* sp.	A2-C1_F	Water	2016-11-10	+	504,641	30.9	4,830,451	36	290,519	50.1	4,406	JAJSOU000000000	SRR17259673
Buttiauxella ferragutiae	A2-C2_NF	Water	2016-11-10	−	549,687	33.7	5,218,071	61	324,474	50.6	4,747	JAJSOV000000000	SRR17259672

Genome sequencing was performed as described previously (8). Preparation of sequencing libraries was performed using a DNA preparation kit (Illumina). Draft genomes were sequenced by 150-bp paired-end sequencing on an Illumina NextSeq 1000 system using NovaGene (Illumina). Reads were trimmed using Cutadapt v1.16.6 (9) and quality controlled using FastQC v0.72 (https://github.com/s-andrews/FastQC). High-quality sequence reads were assembled *de novo* using Unicycler v0.4.6.0 (10), which includes SPAdes v3.12.0 (11). Annotation was carried out using RASTtk v2.0 (12, 13) and NCBI PGAP v5.0 (14, 15). Phylogeny was determined with the codon-tree pipeline in PATRIC v3.6.9 (16, 17), which uses single-copy cross-genus protein families and analyzes aligned proteins and coding DNA from single-copy genes using RAxML v8.0.0 (18). To confirm species, the average nucleotide identity (ANI) was calculated using OrthoANI (19). Default parameters were used for all software unless otherwise noted. Genome sizes and additional information are presented in Table 1.

Phylogenetic analyses and ANI values suggest that three of the isolates (S04-F03, W03-F01, and A2-C1_F) represent a new species of the genus *Buttiauxella*, while the fourth strain (A2-C2_NF) could be assigned to the species Buttiauxella ferragutiae. The analyses further confirmed the presence of the *uidA* gene, coding for β-d-glucuronidase, in the three isolates with fluorescence in the Colilert-18/Quanti-Tray test (S04-F03, W03-F01, and A2-C1_F) and its absence in the fourth isolate (A2-C2_NF). Glucuronidase activity has also been demonstrated in Buttiauxella noackiae MCE (formerly Buttiauxella agrestis), which was isolated from surface water (2). Thus, certain *Buttiauxella* isolates from environmental sources that harbor β-d-glucuronidase can lead to false-positive E. coli results in drinking water testing.

### Data availability.

The whole-genome shotgun projects and the raw sequence reads have been deposited in DDBJ/ENA/GenBank under the accession numbers listed in Table 1. They belong to the BioProject PRJNA789423. For all sequences, the first versions of the accession numbers are described in this paper.
